# Supplementary material for: Generation of a conditional mutant knock-in under the control of the natural promoter using CRISPR-Cas9 and Cre-Lox systems
Source: PLoS One. 2020 Oct 2;15(10):e0240256. doi: 10.1371/journal.pone.0240256 (PMC7531807; doi:10.1371/journal.pone.0240256)
Supplement: S1 Fig — Introns are depicted small letters and exons in capital letters. (PPTX) [file pone.0240256.s001.pptx]

## Slide 1
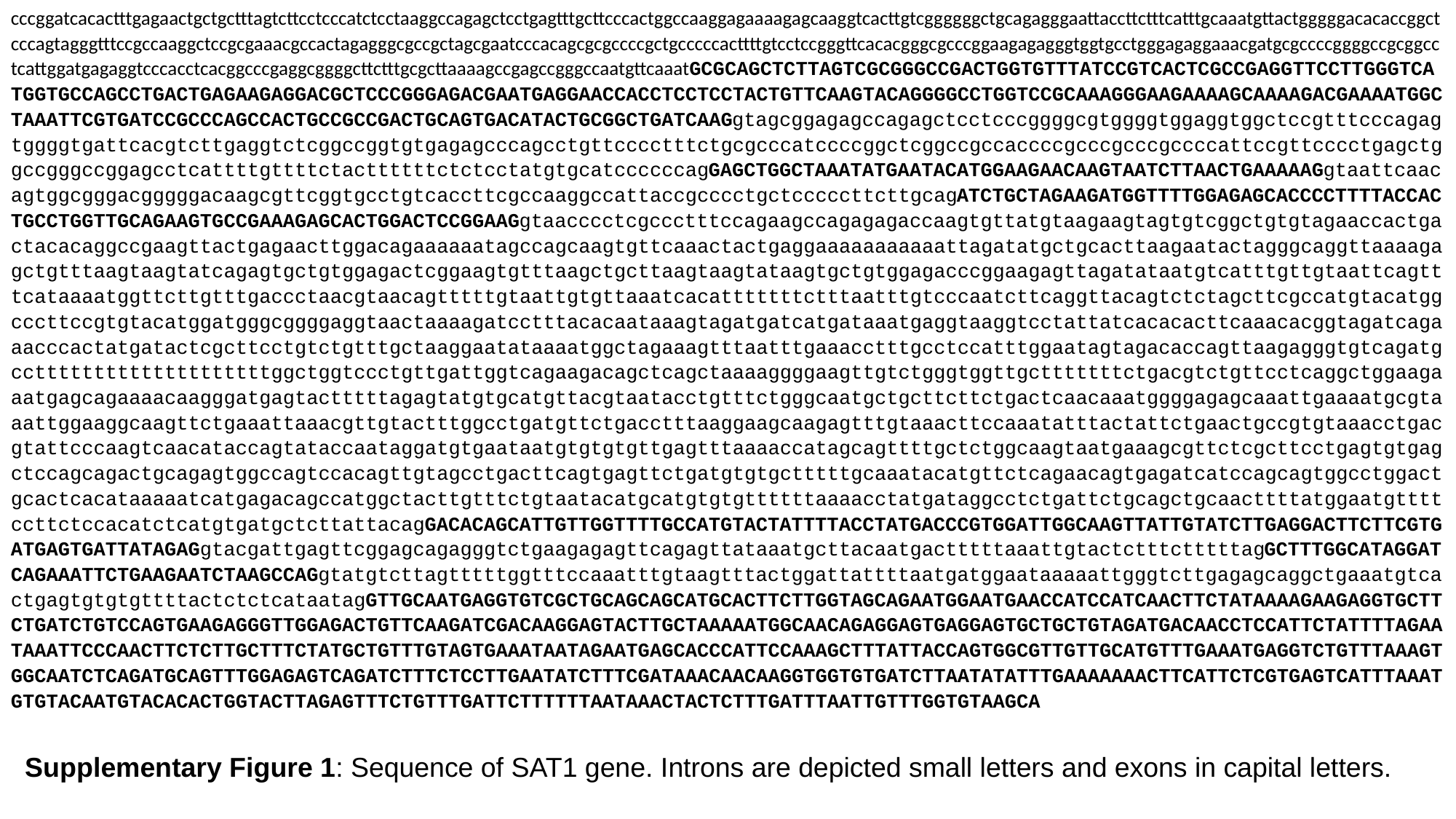

cccggatcacactttgagaactgctgctttagtcttcctcccatctcctaaggccagagctcctgagtttgcttcccactggccaaggagaaaagagcaaggtcacttgtcggggggctgcagagggaattaccttctttcatttgcaaatgttactgggggacacaccggctcccagtagggtttccgccaaggctccgcgaaacgccactagagggcgccgctagcgaatcccacagcgcgccccgctgcccccacttttgtcctccgggttcacacgggcgcccggaagagagggtggtgcctgggagaggaaacgatgcgccccggggccgcggcctcattggatgagaggtcccacctcacggcccgaggcggggcttctttgcgcttaaaagccgagccgggccaatgttcaaatGCGCAGCTCTTAGTCGCGGGCCGACTGGTGTTTATCCGTCACTCGCCGAGGTTCCTTGGGTCATGGTGCCAGCCTGACTGAGAAGAGGACGCTCCCGGGAGACGAATGAGGAACCACCTCCTCCTACTGTTCAAGTACAGGGGCCTGGTCCGCAAAGGGAAGAAAAGCAAAAGACGAAAATGGCTAAATTCGTGATCCGCCCAGCCACTGCCGCCGACTGCAGTGACATACTGCGGCTGATCAAGgtagcggagagccagagctcctcccggggcgtggggtggaggtggctccgtttcccagagtggggtgattcacgtcttgaggtctcggccggtgtgagagcccagcctgttcccctttctgcgcccatccccggctcggccgccaccccgcccgcccgccccattccgttcccctgagctggccgggccggagcctcattttgttttctacttttttctctcctatgtgcatccccccagGAGCTGGCTAAATATGAATACATGGAAGAACAAGTAATCTTAACTGAAAAAGgtaattcaacagtggcgggacgggggacaagcgttcggtgcctgtcaccttcgccaaggccattaccgcccctgctcccccttcttgcagATCTGCTAGAAGATGGTTTTGGAGAGCACCCCTTTTACCACTGCCTGGTTGCAGAAGTGCCGAAAGAGCACTGGACTCCGGAAGgtaacccctcgccctttccagaagccagagagaccaagtgttatgtaagaagtagtgtcggctgtgtagaaccactgactacacaggccgaagttactgagaacttggacagaaaaaatagccagcaagtgttcaaactactgaggaaaaaaaaaaattagatatgctgcacttaagaatactagggcaggttaaaagagctgtttaagtaagtatcagagtgctgtggagactcggaagtgtttaagctgcttaagtaagtataagtgctgtggagacccggaagagttagatataatgtcatttgttgtaattcagtttcataaaatggttcttgtttgaccctaacgtaacagtttttgtaattgtgttaaatcacatttttttctttaatttgtcccaatcttcaggttacagtctctagcttcgccatgtacatggcccttccgtgtacatggatgggcggggaggtaactaaaagatcctttacacaataaagtagatgatcatgataaatgaggtaaggtcctattatcacacacttcaaacacggtagatcagaaacccactatgatactcgcttcctgtctgtttgctaaggaatataaaatggctagaaagtttaatttgaaacctttgcctccatttggaatagtagacaccagttaagagggtgtcagatgccttttttttttttttttttttggctggtccctgttgattggtcagaagacagctcagctaaaaggggaagttgtctgggtggttgctttttttctgacgtctgttcctcaggctggaagaaatgagcagaaaacaagggatgagtactttttagagtatgtgcatgttacgtaatacctgtttctgggcaatgctgcttcttctgactcaacaaatggggagagcaaattgaaaatgcgtaaattggaaggcaagttctgaaattaaacgttgtactttggcctgatgttctgacctttaaggaagcaagagtttgtaaacttccaaatatttactattctgaactgccgtgtaaacctgacgtattcccaagtcaacataccagtataccaataggatgtgaataatgtgtgtgttgagtttaaaaccatagcagttttgctctggcaagtaatgaaagcgttctcgcttcctgagtgtgagctccagcagactgcagagtggccagtccacagttgtagcctgacttcagtgagttctgatgtgtgctttttgcaaatacatgttctcagaacagtgagatcatccagcagtggcctggactgcactcacataaaaatcatgagacagccatggctacttgtttctgtaatacatgcatgtgtgttttttaaaacctatgataggcctctgattctgcagctgcaacttttatggaatgttttccttctccacatctcatgtgatgctcttattacagGACACAGCATTGTTGGTTTTGCCATGTACTATTTTACCTATGACCCGTGGATTGGCAAGTTATTGTATCTTGAGGACTTCTTCGTGATGAGTGATTATAGAGgtacgattgagttcggagcagagggtctgaagagagttcagagttataaatgcttacaatgactttttaaattgtactctttctttttagGCTTTGGCATAGGATCAGAAATTCTGAAGAATCTAAGCCAGgtatgtcttagtttttggtttccaaatttgtaagtttactggattattttaatgatggaataaaaattgggtcttgagagcaggctgaaatgtcactgagtgtgtgttttactctctcataatagGTTGCAATGAGGTGTCGCTGCAGCAGCATGCACTTCTTGGTAGCAGAATGGAATGAACCATCCATCAACTTCTATAAAAGAAGAGGTGCTTCTGATCTGTCCAGTGAAGAGGGTTGGAGACTGTTCAAGATCGACAAGGAGTACTTGCTAAAAATGGCAACAGAGGAGTGAGGAGTGCTGCTGTAGATGACAACCTCCATTCTATTTTAGAATAAATTCCCAACTTCTCTTGCTTTCTATGCTGTTTGTAGTGAAATAATAGAATGAGCACCCATTCCAAAGCTTTATTACCAGTGGCGTTGTTGCATGTTTGAAATGAGGTCTGTTTAAAGTGGCAATCTCAGATGCAGTTTGGAGAGTCAGATCTTTCTCCTTGAATATCTTTCGATAAACAACAAGGTGGTGTGATCTTAATATATTTGAAAAAAACTTCATTCTCGTGAGTCATTTAAATGTGTACAATGTACACACTGGTACTTAGAGTTTCTGTTTGATTCTTTTTTAATAAACTACTCTTTGATTTAATTGTTTGGTGTAAGCA
Supplementary Figure 1: Sequence of SAT1 gene. Introns are depicted small letters and exons in capital letters.
