# Supplementary material for: Generation of a conditional mutant knock-in under the control of the natural promoter using CRISPR-Cas9 and Cre-Lox systems
Source: PLoS One. 2020 Oct 2;15(10):e0240256. doi: 10.1371/journal.pone.0240256 (PMC7531807; doi:10.1371/journal.pone.0240256)
Supplement: S2 Fig — (PPTX) [file pone.0240256.s002.pptx]

## Slide 1
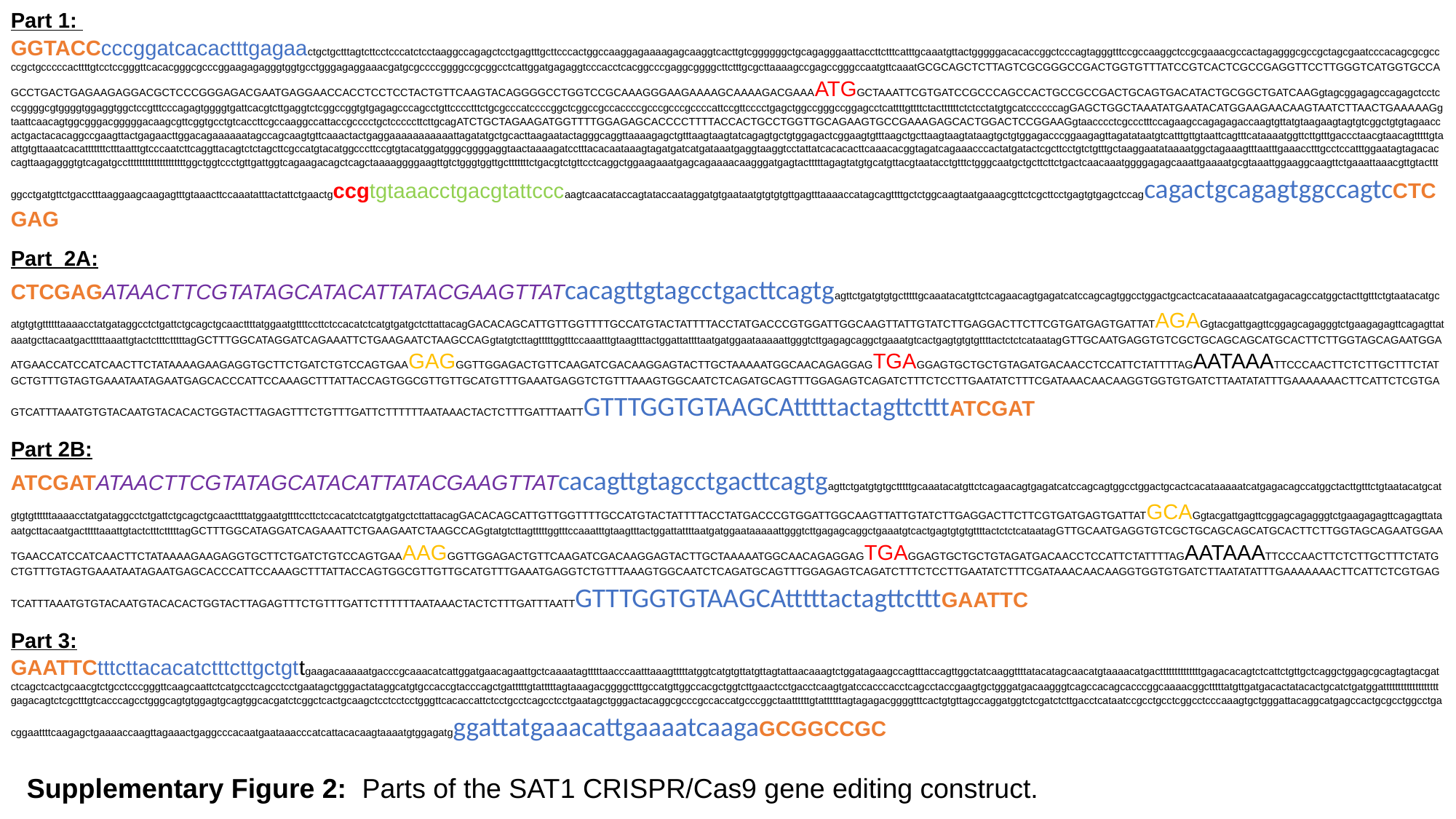

Part 1: GGTACCcccggatcacactttgagaactgctgctttagtcttcctcccatctcctaaggccagagctcctgagtttgcttcccactggccaaggagaaaagagcaaggtcacttgtcggggggctgcagagggaattaccttctttcatttgcaaatgttactgggggacacaccggctcccagtagggtttccgccaaggctccgcgaaacgccactagagggcgccgctagcgaatcccacagcgcgccccgctgcccccacttttgtcctccgggttcacacgggcgcccggaagagagggtggtgcctgggagaggaaacgatgcgccccggggccgcggcctcattggatgagaggtcccacctcacggcccgaggcggggcttctttgcgcttaaaagccgagccgggccaatgttcaaatGCGCAGCTCTTAGTCGCGGGCCGACTGGTGTTTATCCGTCACTCGCCGAGGTTCCTTGGGTCATGGTGCCAGCCTGACTGAGAAGAGGACGCTCCCGGGAGACGAATGAGGAACCACCTCCTCCTACTGTTCAAGTACAGGGGCCTGGTCCGCAAAGGGAAGAAAAGCAAAAGACGAAAATGGCTAAATTCGTGATCCGCCCAGCCACTGCCGCCGACTGCAGTGACATACTGCGGCTGATCAAGgtagcggagagccagagctcctcccggggcgtggggtggaggtggctccgtttcccagagtggggtgattcacgtcttgaggtctcggccggtgtgagagcccagcctgttcccctttctgcgcccatccccggctcggccgccaccccgcccgcccgccccattccgttcccctgagctggccgggccggagcctcattttgttttctacttttttctctcctatgtgcatccccccagGAGCTGGCTAAATATGAATACATGGAAGAACAAGTAATCTTAACTGAAAAAGgtaattcaacagtggcgggacgggggacaagcgttcggtgcctgtcaccttcgccaaggccattaccgcccctgctcccccttcttgcagATCTGCTAGAAGATGGTTTTGGAGAGCACCCCTTTTACCACTGCCTGGTTGCAGAAGTGCCGAAAGAGCACTGGACTCCGGAAGgtaacccctcgccctttccagaagccagagagaccaagtgttatgtaagaagtagtgtcggctgtgtagaaccactgactacacaggccgaagttactgagaacttggacagaaaaaatagccagcaagtgttcaaactactgaggaaaaaaaaaaattagatatgctgcacttaagaatactagggcaggttaaaagagctgtttaagtaagtatcagagtgctgtggagactcggaagtgtttaagctgcttaagtaagtataagtgctgtggagacccggaagagttagatataatgtcatttgttgtaattcagtttcataaaatggttcttgtttgaccctaacgtaacagtttttgtaattgtgttaaatcacatttttttctttaatttgtcccaatcttcaggttacagtctctagcttcgccatgtacatggcccttccgtgtacatggatgggcggggaggtaactaaaagatcctttacacaataaagtagatgatcatgataaatgaggtaaggtcctattatcacacacttcaaacacggtagatcagaaacccactatgatactcgcttcctgtctgtttgctaaggaatataaaatggctagaaagtttaatttgaaacctttgcctccatttggaatagtagacaccagttaagagggtgtcagatgccttttttttttttttttttttggctggtccctgttgattggtcagaagacagctcagctaaaaggggaagttgtctgggtggttgctttttttctgacgtctgttcctcaggctggaagaaatgagcagaaaacaagggatgagtactttttagagtatgtgcatgttacgtaatacctgtttctgggcaatgctgcttcttctgactcaacaaatggggagagcaaattgaaaatgcgtaaattggaaggcaagttctgaaattaaacgttgtactttggcctgatgttctgacctttaaggaagcaagagtttgtaaacttccaaatatttactattctgaactgccgtgtaaacctgacgtattcccaagtcaacataccagtataccaataggatgtgaataatgtgtgtgttgagtttaaaaccatagcagttttgctctggcaagtaatgaaagcgttctcgcttcctgagtgtgagctccagcagactgcagagtggccagtcCTCGAG
Part 2A: CTCGAGATAACTTCGTATAGCATACATTATACGAAGTTATcacagttgtagcctgacttcagtgagttctgatgtgtgctttttgcaaatacatgttctcagaacagtgagatcatccagcagtggcctggactgcactcacataaaaatcatgagacagccatggctacttgtttctgtaatacatgcatgtgtgttttttaaaacctatgataggcctctgattctgcagctgcaacttttatggaatgttttccttctccacatctcatgtgatgctcttattacagGACACAGCATTGTTGGTTTTGCCATGTACTATTTTACCTATGACCCGTGGATTGGCAAGTTATTGTATCTTGAGGACTTCTTCGTGATGAGTGATTATAGAGgtacgattgagttcggagcagagggtctgaagagagttcagagttataaatgcttacaatgactttttaaattgtactctttctttttagGCTTTGGCATAGGATCAGAAATTCTGAAGAATCTAAGCCAGgtatgtcttagtttttggtttccaaatttgtaagtttactggattattttaatgatggaataaaaattgggtcttgagagcaggctgaaatgtcactgagtgtgtgttttactctctcataatagGTTGCAATGAGGTGTCGCTGCAGCAGCATGCACTTCTTGGTAGCAGAATGGAATGAACCATCCATCAACTTCTATAAAAGAAGAGGTGCTTCTGATCTGTCCAGTGAAGAGGGTTGGAGACTGTTCAAGATCGACAAGGAGTACTTGCTAAAAATGGCAACAGAGGAGTGAGGAGTGCTGCTGTAGATGACAACCTCCATTCTATTTTAGAATAAATTCCCAACTTCTCTTGCTTTCTATGCTGTTTGTAGTGAAATAATAGAATGAGCACCCATTCCAAAGCTTTATTACCAGTGGCGTTGTTGCATGTTTGAAATGAGGTCTGTTTAAAGTGGCAATCTCAGATGCAGTTTGGAGAGTCAGATCTTTCTCCTTGAATATCTTTCGATAAACAACAAGGTGGTGTGATCTTAATATATTTGAAAAAAACTTCATTCTCGTGAGTCATTTAAATGTGTACAATGTACACACTGGTACTTAGAGTTTCTGTTTGATTCTTTTTTAATAAACTACTCTTTGATTTAATTGTTTGGTGTAAGCAtttttactagttctttATCGAT
Part 2B: ATCGATATAACTTCGTATAGCATACATTATACGAAGTTATcacagttgtagcctgacttcagtgagttctgatgtgtgctttttgcaaatacatgttctcagaacagtgagatcatccagcagtggcctggactgcactcacataaaaatcatgagacagccatggctacttgtttctgtaatacatgcatgtgtgttttttaaaacctatgataggcctctgattctgcagctgcaacttttatggaatgttttccttctccacatctcatgtgatgctcttattacagGACACAGCATTGTTGGTTTTGCCATGTACTATTTTACCTATGACCCGTGGATTGGCAAGTTATTGTATCTTGAGGACTTCTTCGTGATGAGTGATTATGCAGgtacgattgagttcggagcagagggtctgaagagagttcagagttataaatgcttacaatgactttttaaattgtactctttctttttagGCTTTGGCATAGGATCAGAAATTCTGAAGAATCTAAGCCAGgtatgtcttagtttttggtttccaaatttgtaagtttactggattattttaatgatggaataaaaattgggtcttgagagcaggctgaaatgtcactgagtgtgtgttttactctctcataatagGTTGCAATGAGGTGTCGCTGCAGCAGCATGCACTTCTTGGTAGCAGAATGGAATGAACCATCCATCAACTTCTATAAAAGAAGAGGTGCTTCTGATCTGTCCAGTGAAAAGGGTTGGAGACTGTTCAAGATCGACAAGGAGTACTTGCTAAAAATGGCAACAGAGGAGTGAGGAGTGCTGCTGTAGATGACAACCTCCATTCTATTTTAGAATAAATTCCCAACTTCTCTTGCTTTCTATGCTGTTTGTAGTGAAATAATAGAATGAGCACCCATTCCAAAGCTTTATTACCAGTGGCGTTGTTGCATGTTTGAAATGAGGTCTGTTTAAAGTGGCAATCTCAGATGCAGTTTGGAGAGTCAGATCTTTCTCCTTGAATATCTTTCGATAAACAACAAGGTGGTGTGATCTTAATATATTTGAAAAAAACTTCATTCTCGTGAGTCATTTAAATGTGTACAATGTACACACTGGTACTTAGAGTTTCTGTTTGATTCTTTTTTAATAAACTACTCTTTGATTTAATTGTTTGGTGTAAGCAtttttactagttctttGAATTC
Part 3: GAATTCtttcttacacatctttcttgctgttgaagacaaaaatgacccgcaaacatcattggatgaacagaattgctcaaaatagtttttaacccaatttaaagtttttatggtcatgtgttatgttagtattaacaaagtctggatagaagccagtttaccagttggctatcaaggttttatacatagcaacatgtaaaacatgacttttttttttttttgagacacagtctcattctgttgctcaggctggagcgcagtagtacgatctcagctcactgcaacgtctgcctcccgggttcaagcaattctcatgcctcagcctcctgaatagctgggactataggcatgtgccaccgtacccagctgatttttgtatttttagtaaagacggggctttgccatgttggccacgctggtcttgaactcctgacctcaagtgatccacccacctcagcctaccgaagtgctgggatgacaagggtcagccacagcacccggcaaaacggctttttatgttgatgacactatacactgcatctgatggatttttttttttttttttttgagacagtctcgctttgtcacccagcctgggcagtgtggagtgcagtggcacgatctcggctcactgcaagctcctcctcctgggttcacaccattctcctgcctcagcctcctgaatagctgggactacaggcgcccgccaccatgcccggctaattttttgtattttttagtagagacggggtttcactgtgttagccaggatggtctcgatctcttgacctcataatccgcctgcctcggcctcccaaagtgctgggattacaggcatgagccactgcgcctggcctgacggaattttcaagagctgaaaaccaagttagaaactgaggcccacaatgaataaacccatcattacacaagtaaaatgtggagatgggattatgaaacattgaaaatcaagaGCGGCCGC
Supplementary Figure 2: Parts of the SAT1 CRISPR/Cas9 gene editing construct.

## Slide 2
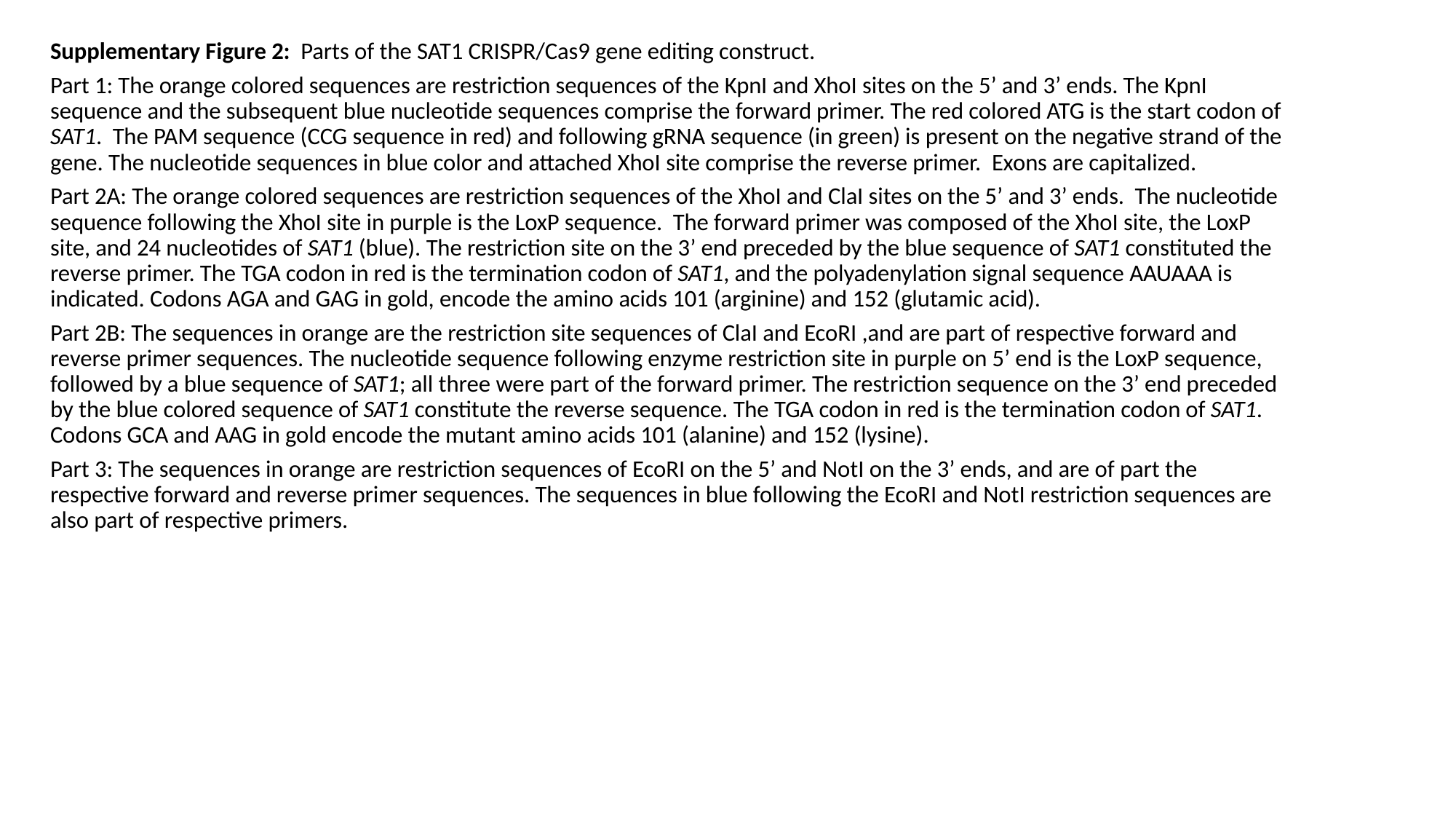

Supplementary Figure 2: Parts of the SAT1 CRISPR/Cas9 gene editing construct.
Part 1: The orange colored sequences are restriction sequences of the KpnI and XhoI sites on the 5’ and 3’ ends. The KpnI sequence and the subsequent blue nucleotide sequences comprise the forward primer. The red colored ATG is the start codon of SAT1. The PAM sequence (CCG sequence in red) and following gRNA sequence (in green) is present on the negative strand of the gene. The nucleotide sequences in blue color and attached XhoI site comprise the reverse primer. Exons are capitalized.
Part 2A: The orange colored sequences are restriction sequences of the XhoI and ClaI sites on the 5’ and 3’ ends. The nucleotide sequence following the XhoI site in purple is the LoxP sequence. The forward primer was composed of the XhoI site, the LoxP site, and 24 nucleotides of SAT1 (blue). The restriction site on the 3’ end preceded by the blue sequence of SAT1 constituted the reverse primer. The TGA codon in red is the termination codon of SAT1, and the polyadenylation signal sequence AAUAAA is indicated. Codons AGA and GAG in gold, encode the amino acids 101 (arginine) and 152 (glutamic acid).
Part 2B: The sequences in orange are the restriction site sequences of ClaI and EcoRI ,and are part of respective forward and reverse primer sequences. The nucleotide sequence following enzyme restriction site in purple on 5’ end is the LoxP sequence, followed by a blue sequence of SAT1; all three were part of the forward primer. The restriction sequence on the 3’ end preceded by the blue colored sequence of SAT1 constitute the reverse sequence. The TGA codon in red is the termination codon of SAT1. Codons GCA and AAG in gold encode the mutant amino acids 101 (alanine) and 152 (lysine).
Part 3: The sequences in orange are restriction sequences of EcoRI on the 5’ and NotI on the 3’ ends, and are of part the respective forward and reverse primer sequences. The sequences in blue following the EcoRI and NotI restriction sequences are also part of respective primers.
