# Supplementary material for: Generation of a conditional mutant knock-in under the control of the natural promoter using CRISPR-Cas9 and Cre-Lox systems
Source: PLoS One. 2020 Oct 2;15(10):e0240256. doi: 10.1371/journal.pone.0240256 (PMC7531807; doi:10.1371/journal.pone.0240256)
Supplement: S3 Fig — Addition or deletion on 5’ and 3’ end of the insert on DNA sequence. (PPTX) [file pone.0240256.s003.pptx]

## Slide 1
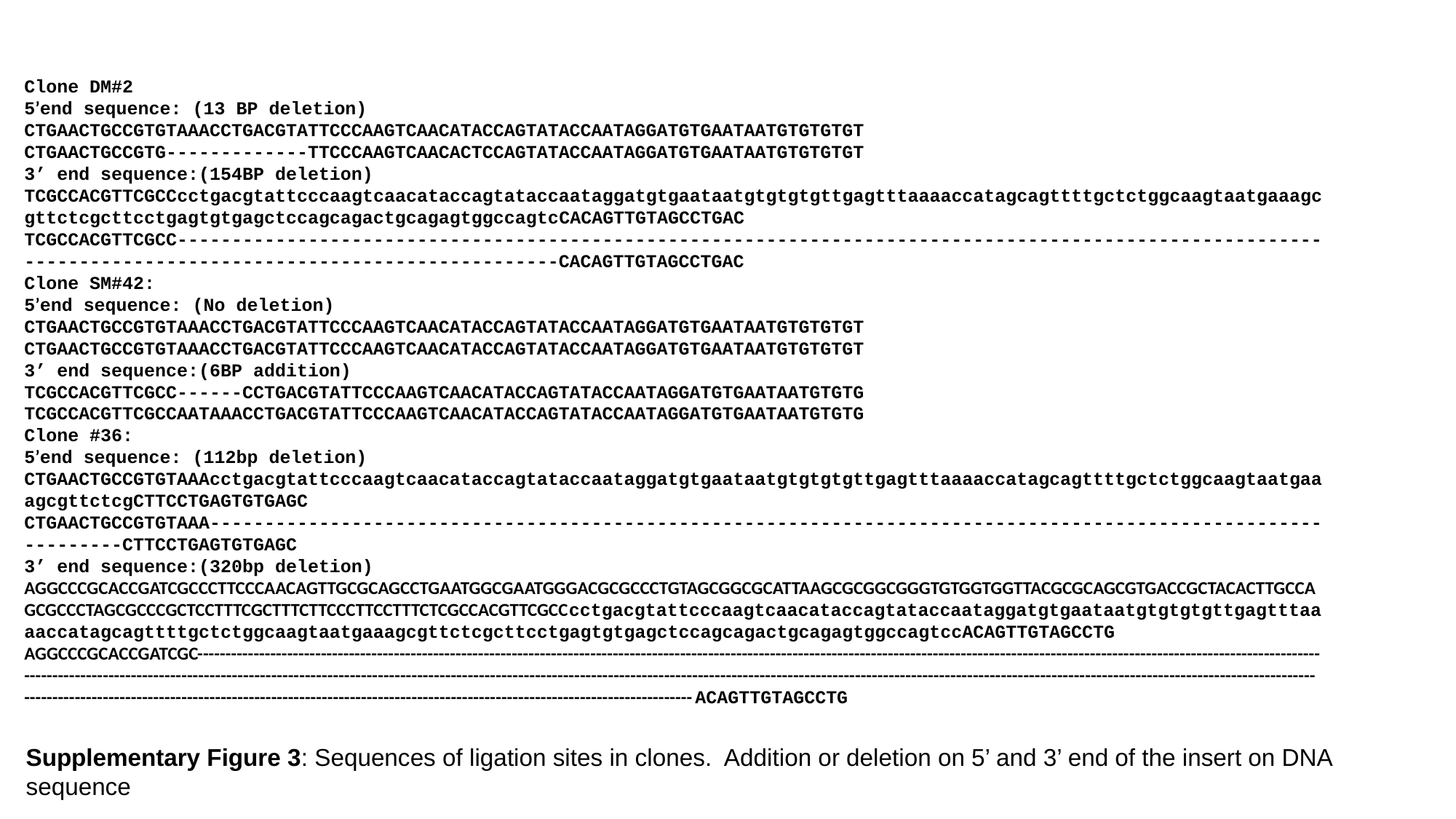

Clone DM#2
5’end sequence: (13 BP deletion)
CTGAACTGCCGTGTAAACCTGACGTATTCCCAAGTCAACATACCAGTATACCAATAGGATGTGAATAATGTGTGTGT
CTGAACTGCCGTG-------------TTCCCAAGTCAACACTCCAGTATACCAATAGGATGTGAATAATGTGTGTGT
3’ end sequence:(154BP deletion)
TCGCCACGTTCGCCcctgacgtattcccaagtcaacataccagtataccaataggatgtgaataatgtgtgtgttgagtttaaaaccatagcagttttgctctggcaagtaatgaaagcgttctcgcttcctgagtgtgagctccagcagactgcagagtggccagtcCACAGTTGTAGCCTGAC
TCGCCACGTTCGCC----------------------------------------------------------------------------------------------------------------------------------------------------------CACAGTTGTAGCCTGAC
Clone SM#42:
5’end sequence: (No deletion)
CTGAACTGCCGTGTAAACCTGACGTATTCCCAAGTCAACATACCAGTATACCAATAGGATGTGAATAATGTGTGTGT
CTGAACTGCCGTGTAAACCTGACGTATTCCCAAGTCAACATACCAGTATACCAATAGGATGTGAATAATGTGTGTGT
3’ end sequence:(6BP addition)
TCGCCACGTTCGCC------CCTGACGTATTCCCAAGTCAACATACCAGTATACCAATAGGATGTGAATAATGTGTG
TCGCCACGTTCGCCAATAAACCTGACGTATTCCCAAGTCAACATACCAGTATACCAATAGGATGTGAATAATGTGTG
Clone #36:
5’end sequence: (112bp deletion)
CTGAACTGCCGTGTAAAcctgacgtattcccaagtcaacataccagtataccaataggatgtgaataatgtgtgtgttgagtttaaaaccatagcagttttgctctggcaagtaatgaaagcgttctcgCTTCCTGAGTGTGAGC
CTGAACTGCCGTGTAAA---------------------------------------------------------------------------------------------------------------CTTCCTGAGTGTGAGC
3’ end sequence:(320bp deletion)
AGGCCCGCACCGATCGCCCTTCCCAACAGTTGCGCAGCCTGAATGGCGAATGGGACGCGCCCTGTAGCGGCGCATTAAGCGCGGCGGGTGTGGTGGTTACGCGCAGCGTGACCGCTACACTTGCCAGCGCCCTAGCGCCCGCTCCTTTCGCTTTCTTCCCTTCCTTTCTCGCCACGTTCGCCcctgacgtattcccaagtcaacataccagtataccaataggatgtgaataatgtgtgtgttgagtttaaaaccatagcagttttgctctggcaagtaatgaaagcgttctcgcttcctgagtgtgagctccagcagactgcagagtggccagtccACAGTTGTAGCCTG
AGGCCCGCACCGATCGC---------------------------------------------------------------------------------------------------------------------------------------------------------------------------------------------------------------------------------------------------------------------------------------------------------------------------------------------------------------------------------------------------------------------------------------------------------------------------------------------------------------------------------------------------------------------ACAGTTGTAGCCTG
Supplementary Figure 3: Sequences of ligation sites in clones. Addition or deletion on 5’ and 3’ end of the insert on DNA sequence
