# Supplementary figures and images for: Generation of a conditional mutant knock-in under the control of the natural promoter using CRISPR-Cas9 and Cre-Lox systems
Source: PLoS One. 2020 Oct 2;15(10):e0240256. doi: 10.1371/journal.pone.0240256 (PMC7531807; doi:10.1371/journal.pone.0240256)

## Slide 1
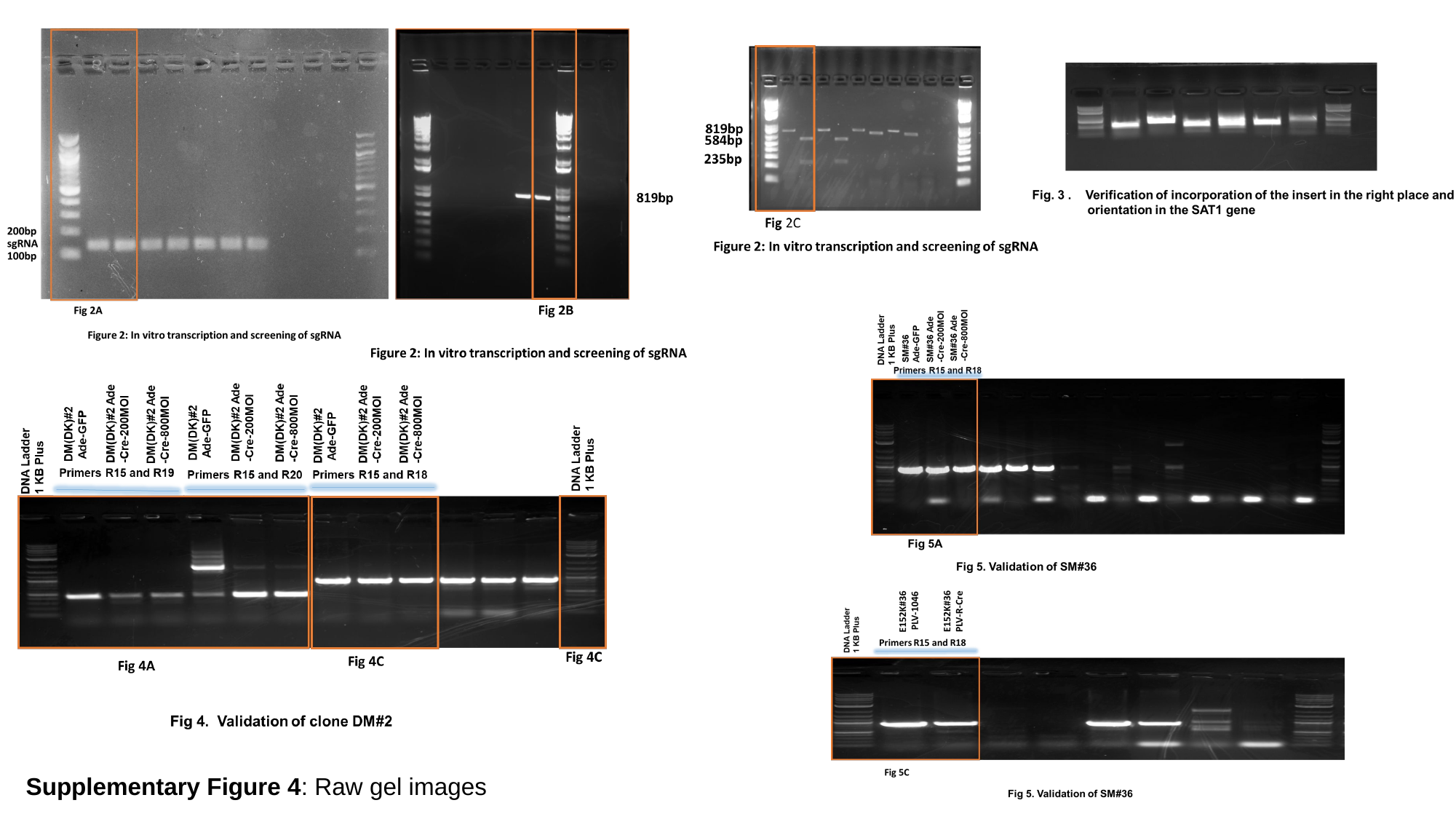

Supplementary Figure 4: Raw gel images

Supplement: S4 Fig — (PPTX) [file pone.0240256.s004.pptx]

## Slide 1
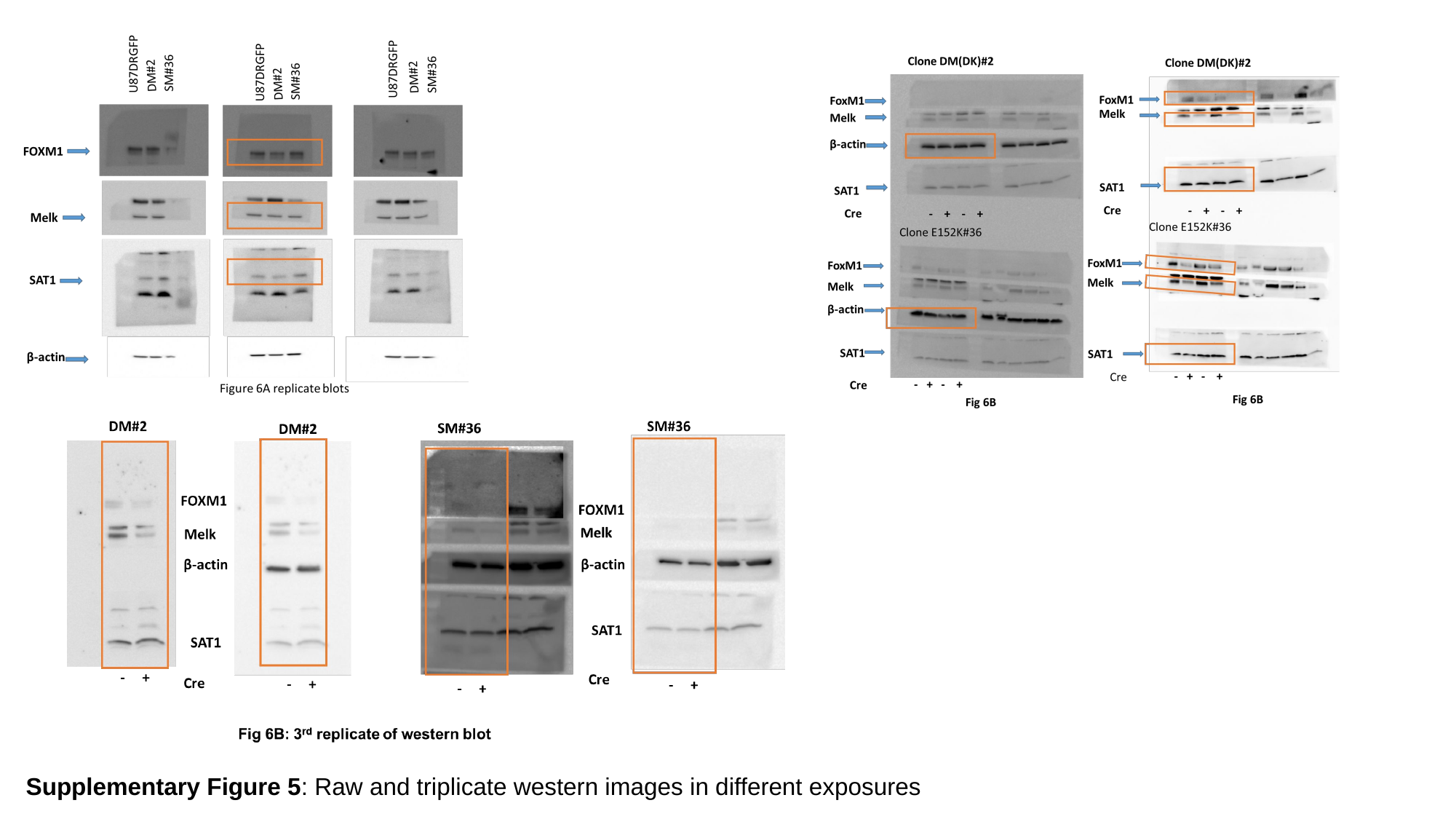

Supplementary Figure 5: Raw and triplicate western images in different exposures

Supplement: S5 Fig — (PPTX) [file pone.0240256.s005.pptx]
