# Supplementary material for: Generation of a conditional mutant knock-in under the control of the natural promoter using CRISPR-Cas9 and Cre-Lox systems
Source: PLoS One. 2020 Oct 2;15(10):e0240256. doi: 10.1371/journal.pone.0240256 (PMC7531807; doi:10.1371/journal.pone.0240256)
Supplement: Attachment — Submitted filename: responses to reviewers.docx [file pone.0240256.s006.docx]

**AE:**

- Experimental and Statistical.
  - Present the raw data for Western blots in supplementary experiments.

Raw data now presented in supplementary figure 4 and 5.

- Provide a link to all of the sequencing data.

All sequencing data are presented in supplementary figure 3.

- Provide the raw data for Western blots and all other electrophoretic experiments.

Raw data now presented in supplementary figure 4 and 5.

- As noted by Reviewer 2, the Western blots of Sat1 and its targets must be compared with the original cell line.

New data has been added to figure 6 to address this.

- Please indicate the number of times the Western analysis was performed, quantify the data, and determine statistical significance. If the experimental has not been repeated, please conduct at least three times (including the above controls).

All westerns were performed three times, quantified, and presented now in figure 6 and supplementary 5.

- Textual
  - In the Results section, 1 st paragraph what is the meaning of "score"

The gRNA design tool CHOPCHOP was used, and a high scoring gRNA with minimal predicted off target sites was chosen. We have clarified this in the text.

- The Results section should open with a brief overview of the method that should be shown diagrammatically in a new Figure 1.

A new Figure 1A has been made, and appropriately introduced.

- The Materials and Methods are incomplete and lack methods including electrophoretic and blotting techniques such as Western analysis as sequencing and statistical analysis (for Westerns).

More details were added.

- The text contains multiple grammatical errors throughout.  Please correct these errors. (Examples: Abstract:"an identical region SAT1 containing";  "CRISPR/Cas9 induced DNA double break".)

Completed.

- Please provide more detail in the current Figure 1 as suggested by Reviewer 3.

Completed.

**Reviewer #1:** 

Minor issues to be addressed:
1. Are there any published approaches that combining CRISPR/Cas9 and Cre-Lox systems, either using in mammalian or in other eukaryotic cells? It would be valuable for the readers to know the differences between the existing methods and the one established in this study.

Indeed, there is a published approach that combined CRISPR/Cas9 and Cre-Lox systems and has been cited in the paper (reference 7). However, in that study, WT gene was not modified, but an exogenous gene under an artificial promoter was introduced which can then be manipulated using Cre-Lox system. In our paper, we modified the WT SAT1 gene such that it can be converted to its mutated version by cre activation while still under its natural promoter.

2. Please give the detail data about the efficiency and the positive rate of this method in the text.

This data has been added to the results.

3. Please give a clear information of “U87MG cells” in the text.

U87MG has been properly introduced.

4. In figure 1, please explain “BSD” and “hPGK” in the figure legend.

The abbreviations have been added to the legend.

**Reviewer #2:**
1)How many clones where tested to find the two ones that have the correct integrations and are further characterized?

This data is added to the results.

2)it would be much easier to evaluate the PCR results if the primer position would be indicated in a schematic like the one in fig 1B-C.

Primer locations were added to Figure 1A

3)The western blot analysis of Sat1 and its targets should be integrated with a comparison of the same levels in the cell lines from which the transgenic ones were derived (U87MG) to show that the cassette integration doesn't affect the expression and is regulated at endogenous levels.

This data was now added to figure 6.

4)The western blots should be performed in triplicates at least and the results quantified as it's common standard nowadays. Also Uncropped blots should be made available.

All westerns were performed three times, quantified and the data are presented in figure 6 and in supplementary figure 5

Minor point
The file "paper tables" contains also the supplementary figures that are therefore duplicated.

The duplication was removed.

**Reviewer #3:**
1. Supplementary Figure 1 and 2 should be summarized as a figure and put in Figure 1. The scheme should include start codon, stop codon, mutation sites etc.

A new panel was added to figure 1.

2. Stop codon should be marked in both WT and mutant SAT1 in Figure 1.

Stop codons are identified.

3. Figure 4 and 5, the location of primers should be labeled in a scheme of the transgene.

Primer locations were added to Figure 1A

4. Figure 4C and D are not described in the text.

The text was amended to include the references to Figure 4C and D

5. Figure 6, additional SAT1 activity assays would strengthen the conclusion.

We agree that activity assays would strengthen the biology behind the story here, but felt this was a bit beyond the scope of the study. As we are not claiming a biological advance in this work, the sequencing confirms the presence of the alteration. We do appreciate the comment and are applying this to future studies currently.

6. Off-target integrations occurred during the NHEJ-mediated knock-in were discussed, but not studied.

There is of course always a chance that off target editing will occur. Because we saw no changes to the endogenous levels of SAT1 before Cre recombinase activity excised the loxP cassette, we are confident that off target effects are likely minimal in our case. A comment was added to the results to address this point.

7. Limitations of this new approach should be discussed.

The discussion was amended to address limitations.
